# The role of life satisfaction and living arrangements in the association between chronic disease and depression: a national cross-sectional survey

**DOI:** 10.3389/fpsyg.2023.1266059

**Published:** 2023-10-27

**Authors:** Zhao Shang, Yuqing Liu, Dongyu Xue, Yiping Zheng, Yueping Li, Baoquan Zhang, Yue Dai

**Affiliations:** ^1^School of Health Management, Fujian Medical University, Fuzhou, Fujian, China; ^2^Fujian Provincial Maternity and Children’s Hospital, Fuzhou, Fujian, China

**Keywords:** life satisfaction, living arrangements, chronic diseases, depressive symptoms, mediating effect, moderating effect

## Abstract

**Introduction:**

For middle-aged and older people, depression is a frequent and prevalent illness. The purpose of this study was to examine the moderating function of living arrangements in the mediating model as well as the mediating role of life satisfaction in the association between chronic diseases and depressive symptoms.

**Methods:**

The China Health and Retirement Longitudinal Study (CHARLS) provided the data for this investigation (2018). Respondents were grouped according to depression status to compare the differences between middle-aged and older people with different depression statuses. The moderating effect of living arrangements and the mediating effect of life satisfaction were tested using the Bootstrap program and the simple slope approach.

**Results:**

The population’s total prevalence of depressive symptoms was 30.3%. According to the mediating effect research, middle-aged and older people with chronic diseases experienced substantial direct effects on depressive symptoms (*β* = 1.011, *p* < 0.001). It has been established that life satisfaction has an 18.6% mediation effect between depressive symptoms and chronic diseases. Regarding the further moderating influence, it was discovered that chronic diseases had a more significant impact on the life satisfaction of middle-aged and older people who are in live alone than those who are living with others (*β* = 0.037, *p* < 0.05).

**Conclusion:**

In middle-aged and older people, chronic diseases have a major influence on depressive symptoms. Life satisfaction mediated the relationship between chronic diseases and depressive symptoms, and living arrangements moderated the first part of the route in the mediation model. Therefore, life satisfaction and living arrangements should be important considerations to decrease the prevalence of depressive symptoms in middle-aged and older people.

## Introduction

The critical public health problem of depression has become a significant issue for middle-aged and older people’s mental health. Depression symptoms affect around 32 million individuals globally (World Health Organization, 2017). By 2030, it is predicted to be the main contributor to the burden of illness worldwide (Malhi and Mann, 2018). With the aging of the population, depression in the middle-aged and elderly will become relatively common. According to Li et al. (2021) 32.62% of middle-aged and older people reported having depression in 2015 and research from the China Health and Retirement Longitudinal Survey of 2018 suggested that 42.92% of middle-aged and older people in China suffer from depression (Wang et al., 2022), we can see that depression is becoming more common among middle-aged and older people. It not only lowers one’s quality of life and social functioning, but it also frequently coexists with some prevalent ailments. It is clear that in order to contribute to the effective prevention and treatment of depression, it is essential to identify the influencing factors by which middle-aged and older people experience depressive symptoms and study the link between them and depressive symptoms.

Chronic disease can impair middle-aged and older people’s self-assessed health and cognitive function, resulting in bodily dysfunction that can precipitate and worsen the start and progression of geriatric depression. A prior study found that those who had numerous medical illnesses (co-occurrence of two or more chronic noncommunicable diseases) were two to three times more likely to develop depression than people who did not have multiple medical conditions or chronic illnesses (Read et al., 2017). Chronic disease duration, the need for long-term medicine, persistent comorbidities, poor physical performance, an excessive financial burden, and a lack of spiritual support can all increase depression symptoms in older persons (Ma et al., 2015). Another study suggests a bidirectional relationship between depression and chronic disease (Pan et al., 2012). Furthermore, according to the World Health Organization’s (WHO) Global Burden of Disease research, people with chronic conditions are far more likely to suffer from depression. As a result, having a chronic disease raises the risk of developing depression symptoms.

Life satisfaction is a crucial aspect of subjective well-being and is associated with judging an individual’s quality of life (Shad et al., 2017). Patients with chronic diseases have poor health and reduced capacity to complete activities of daily life (Violán et al., 2016). A previous study showed that poor health affects the decline in life satisfaction (Chokkanathan and Mohanty, 2017). Wister et al. found that multimorbidity and chronic diseases are linked to poorer satisfaction levels (Wister et al., 2016). According to certain research, chronic respiratory illness patients had reduced life satisfaction (Hu et al., 2016; Drop et al., 2018; Tanrıverdi and Korkmaz, 2021). Additionally, another study revealed a link between poorer life satisfaction and cardiovascular disease risk factors among African-Americans participating in longitudinal research (Mendez et al., 2018). Life satisfaction is a factor in mental health and quality of life (Rosella et al., 2019). In individuals with cardiovascular disease, a strong link between life satisfaction and depression has been found (Steca et al., 2013). A previous study revealed that people with lower life satisfaction tend to be more likely to experience depressive symptoms (Mei et al., 2021), Smorti et al. found an association between depression, anxiety, and life satisfaction in the research of amyloid light chain cardiac amyloidosis (Smorti et al., 2016). Decreased life satisfaction can affect well-being, there is a risk of bad mood and subsequent development of depression or anxiety. Consequently, chronic diseases may cause reduced life satisfaction and lead to depressive symptoms due to poor mood and anxiety.

The treatment of depressive symptoms involves both personal and external environmental factors. Personal behavior modifications are the outcome of interactions between an individual and their environment (Anderies, 2014). The living arrangement reflects the structure of the family and the way family life is organized, and the structural level determines the way family members interact (Gao and Yao, 2020). Previous studies have revealed that compared to older people living with other adults, those who live alone have a greater frequency of serious chronic diseases (Park et al., 2014; Srivastava et al., 2021), a meta-analysis shows that older adults who live alone are more likely to experience depression (Xiu-Ying et al., 2012). Because of things like family structure and connections, a study has shown that older people who live with their spouse and children have greater life satisfaction than those who live alone (Kooshiar et al., 2012). Kandapan et al. found that older people’s living arrangements in India substantially impacted how satisfied they felt with their lives (Kandapan et al., 2023).

**Figure 1:**
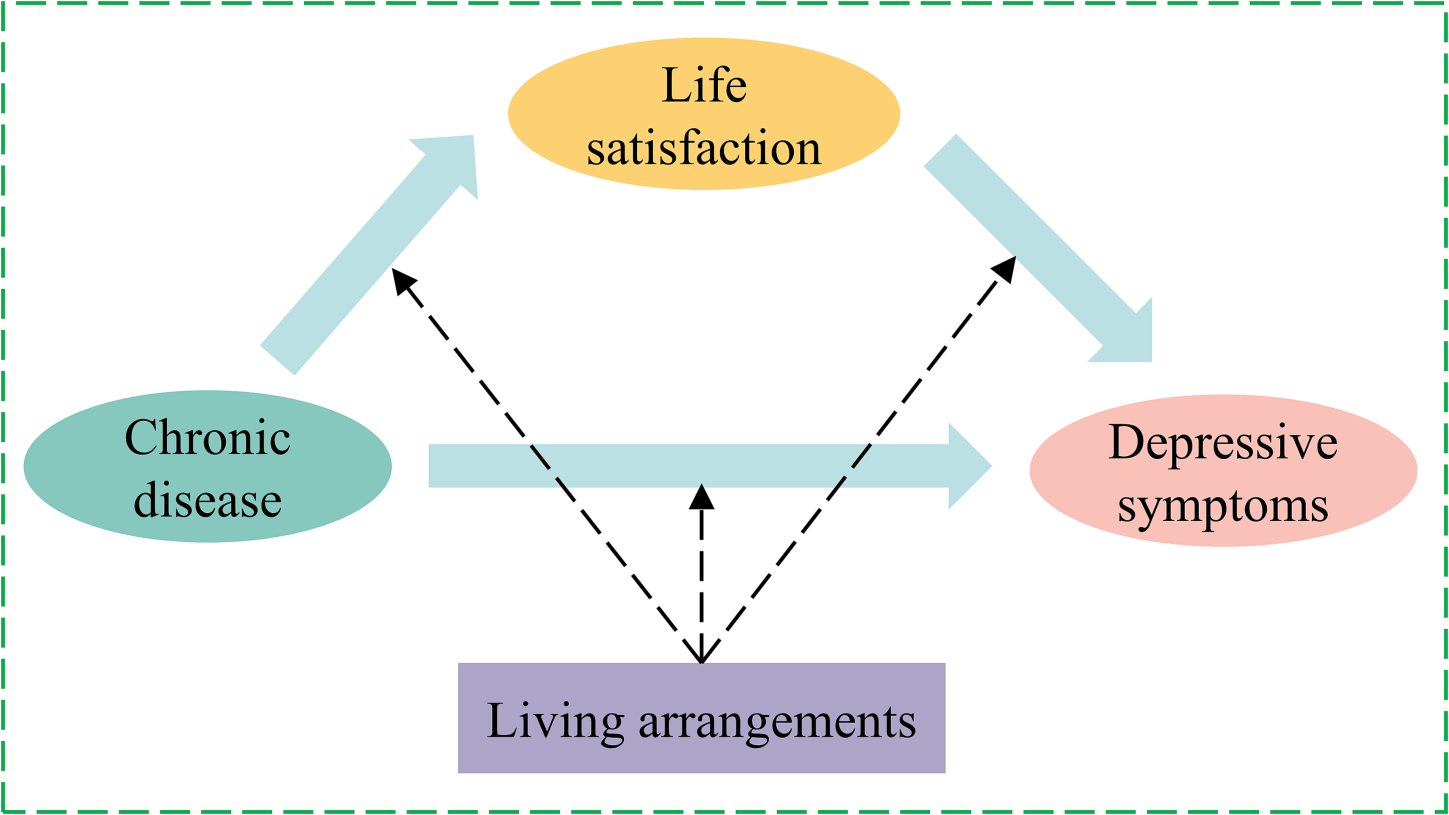
Logic framework diagram.

Based on the facts mentioned above, earlier research has examined the direct relationships between depressive symptoms and chronic diseases, the direct impact of living arrangements and life satisfaction on chronic disease, and depressive symptoms. Current research is sparse on how the living arrangements and life satisfaction of middle-aged and older adults affect the relationship between chronic illness and depression. Given the rising rate of multimorbidity, it is crucial to evaluate this relationship to determine how to prevent and treat depression. Depressive symptoms and mental health in middle-aged and older people may be improved by looking at the relationship between chronic diseases and depressive symptoms in middle-aged and older people and their mechanisms from the perspectives of life satisfaction and living arrangements. We provide three hypotheses for this investigation in light of those above, as seen in Figure 1.

H1: Chronic diseases directly impact middle-aged and older people’s depressive symptoms.

H2: Through the intermediate effect of life satisfaction, chronic diseases directly affect depressive symptoms in middle-aged and older people.

H3: Living arrangements moderate the mediating model of life satisfaction.

## Materials and methods

### Participants and procedure

**Figure 2:**
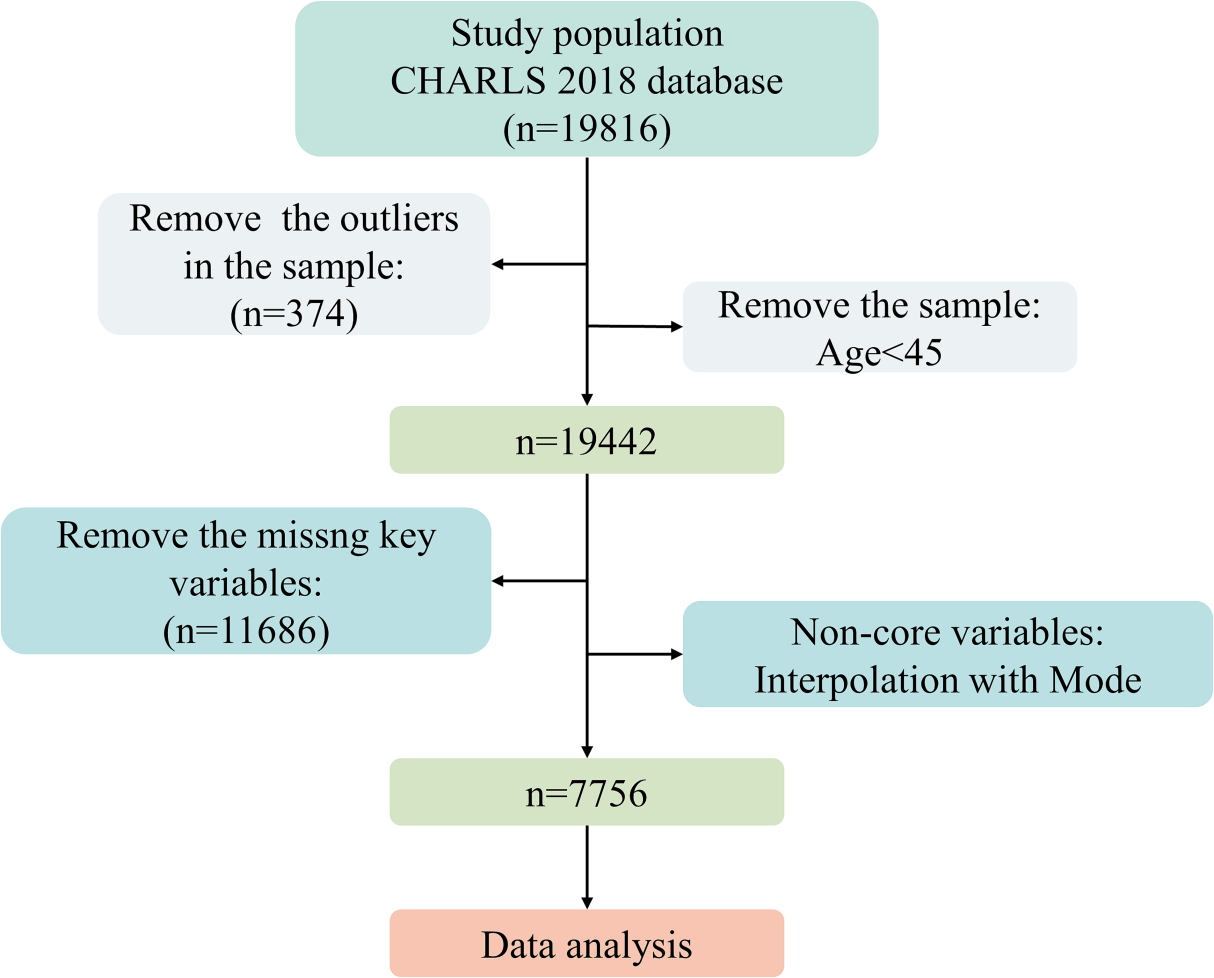
Flowchart of the sample selection procedure.

This study used the latest data from wave 4 (2018) published on the official website of the China Health and Retirement Longitudinal Study (CHARLS). The goal of CHARLS is to gather a set of high-quality microdata typical of Chinese families and people at least 45 years old and in their middle years, 19,816 respondents in a total of 12,400 households in 2018, In this study, We removed 374 outliers and ages<45, leaving 19,442 samples. We excluded samples with missing key variables (life satisfaction, living arrangements) and non-core variables (smoking, drinking) interpolation is performed using the mode, with a final sample size of 7,756 (Figure 2).

## Measures

### Socio-demographic variables

The socio-demographic variables considered in the analysis were gender (male/female), age, marital status (married/unmarried), education level (middle school and below/high school and above), annual household income (<100,000¥,100,000¥-, 300000¥-,500000¥-), living arrangements (living alone/living with other people). Smoking statuses (smoking/no smoking) and drinking statuses (drinking/no drinking) were included in the category of health behaviors.

### Chronic diseases condition

The CHARLS database asks the middle-aged and elderly separately if they have any of the 14 chronic diseases informed by their physicians. They are defined as having a chronic disease if they have at least one condition. Each chronic disease was assigned 1 point, with higher scores indicating a greater number of chronic diseases.

### Depressive symptoms

The CHARLS program uses an epidemiological survey measured by the Depression Scale (CES-D). CES-D scale options consist of 4 levels: “rarely or not at all = 0,” “not too much =1,” “sometimes or half of the time = 2,” and “most of the time = 3,” with a composite score range of 0 to 30. Depressive symptoms were thought to be present in the CES-D ≥ 10 (Chen and Mui, 2014) and classified as the “depressed group”; CES-D < 10 was supposed to have no depressive symptoms and classified as a “non-depressed group,” the Cronbach’s Alpha coefficient of the CES-D scale used in CHARLS was 0. 795, and its reliability met the requirements for statistical analysis.

### Life satisfaction

Life Satisfaction comes from the CHARLS survey question “Overall, are you satisfied with your life? “there are five options to answer: extremely satisfied, very satisfied, relatively satisfied, not very satisfied, and not satisfied at all, respectively, with values of 5, 4, 3, 2, and 1. The higher the score, the higher the life satisfaction.

### Data analysis

IBM SPSS (version 26.0) was employed to analyze data. According to the depressive symptoms score, the study participants were divided into groups and conducted a single-factor analysis. If the measures did not fit the normal distribution, the median was used, and the Mann–Whitney *U* test was used to compare the differences between groups; otherwise, $\bar{X}\pm S$ was used, and the *t*-test was used to compare the differences between groups. For statistical data, the number of cases (percentage) was used, and the *χ^2^* test was used to compare the differences between groups. The Spearman correlation was utilized to investigate the relationship between the research variables. Mediation effect test using bias-corrected percentile Bootstrap method, improves the statistical test power. The design of the specific model is as follows:


(1) $$Y=i+cX+e_{1}$$

(2) $$M=i+\mathrm{aX}+e_{2}$$

(3) $$Y=i+c^{\prime }X+bM+e_{3}$$

In the model, Y is the dependent variable depressive symptoms, X is the independent variable chronic illness, and M is the mediating variable life satisfaction. The specific mediation effect test procedure is as follows: if a × b is significant, the mediation test result does not contain 0 at the 95% confidence interval, the mediation path exists. Conversely, a mediation effect is not significant if the 95% confidence interval of the mediation effect value contains 0. if c’ is not significant, then it is a full mediation effect, if c’ is significant then it is a partial mediation effect. If a × b is not significant, the mediator does not hold and the test is continued to see if c’ is significant, if c’ is significant, it has only a direct effect, and if c’ is not significant, it has no effect. Moderated effects were tested using the bias-corrected percentile Bootstrap method, with chronic diseases as the independent variable, depressive symptoms as the dependent variable, life satisfaction as the mediator, the product of the above variables is the interaction term for the moderating effect. When the moderating effect is significant, the simple slope test suggested by Aiken and West (1991) was employed to examine the precise moderating impact of different living arrangements.

Model 4 and Model 59 in PROCESS 3.4 developed by Hayes (2013) were used to analyze the mediating effect of life satisfaction and the moderating effect of living arrangements. A sample of 5,000 Bootstrap samples as taken and a confidence interval of 95% (95% CI) was set. The two models included adjustments for the relevant covariates (gender, age, marital status, education level, annual household income, smoking, and drinking), which may impact depressive symptoms. The statistically significant level was set at α = 0.05.

## Results

### Baseline comparison between the middle-aged and elderly depressed patient group and the non-depressed patient group

In our study, a total of 7,756 middle-aged and older people were included. The population was divided into groups based on the CES-D ≥ 10, and a single-factor analysis was carried out (Table 1). The results showed that 2,348 (30.3%) individuals had depressive symptoms. No statistically significant differences were found for depressive symptoms by age, living arrangement, smoking status, and drinking status (*p* > 0.05), however, there were statistically significant differences for different gender, education level, annual household income, marital status, number of chronic diseases, and life satisfaction (*p* < 0.001).

**Table 1:** Comparison of middle-aged and older people with depression group and without depression group at baseline.

Variables	CES-D		*χ^2^*/Z
	<10 (*n* = 5,408)	≥10 (*n* = 2,348)	
Gender			149.970***
Male	2,905 (46.3)	906 (36.6)	
Female	2,503 (53.7)	1,442 (38.6)	
Age	54.0 (50.0,60.0)	54.0 (51.0,60.0)	1.786
Marital status			15.805***
Married	5,198 (96.1)	2,209 (94.1)	
Unmarried	210 (5.9)	139 (5.9)	
Education level			79.787***
Middle school and below	4,354 (80.5)	2085 (88.8)	
High school and above	1,054 (19.5)	263 (11.2)	
Annual household income			39.763***
<100,000¥	4,965 (91.8)	2,246 (95.7)	
100,000¥-	406 (7.5)	97 (4.1)	
300,000¥-	29 (0.5)	2 (0.1)	
500,000¥-	8 (0.1)	3 (0.1)	
Living arrangements			0.866
Living alone	2,598 (48.0)	1,101 (46.9)	
Living with other people	2,810 (52.0)	1,247 (53.1)	
Smoking status			1.051
Smoking	281 (5.2)	109 (4.6)	
No smoking	5,127 (94.8)	2,239 (95.4)	
Drinking status			0.166
Drinking	4,925 (91.1)	2,145 (91.4)	
No drinking	483 (8.9)	203 (8.6)	
Number of chronic diseases			114.906***
0	3,364 (62.2)	1,183 (50.4)	
1	1,365 (25.2)	689 (29.3)	
≥2	679 (12.6)	476 (20.3)	
Life satisfaction	3.0 (3.0,4.0)	3.0 (2.0,3.0)	−24.791***

***p < 0.001.

### Correlation analysis and covariance diagnosis

According to the Spearman correlation analysis results, the number of chronic diseases was significantly correlated with life satisfaction and depressive symptoms (*r* = −0.08, *p* < 0.01; *r* = 0.15, *p* < 0.01). In addition, a negative correlation (*r* = −0.34, *p* < 0.01) was found between life satisfaction and depressive symptoms. The requirement of the mediating influence analysis is satisfied by this finding (Table 2). The research identified covariates for the variables in the regression analysis. (Table 3), the results showed that the variance inflation factors for these variables were less than 5, suggesting that none of these variables has covariance.

**Table 2:** Correlation analysis of chronic diseases, life satisfaction, and depressive symptoms.

Variables	1. Chronic diseases	2. Life satisfaction	3. Depressive symptoms
1. Chronic diseases	1.00		
2. Life satisfaction	−0.08**	1.00	
3. Depressive symptoms	0.15**	−0.34**	1.00

**p < 0.01.

**Table 3:** Covariance diagnosis.

Variables	Variance inflation factor
Gender	1.073
Age	1.031
Marital status	1.017
Education level	1.070
Annual household income	1.064
Smoking	1.056
Drinking	1.009
Life satisfaction	1.015
Chronic diseases	1.024
Living arrangements	1.008

### Mediating effect of life satisfaction between chronic diseases and depressive symptoms

**Figure 3:**
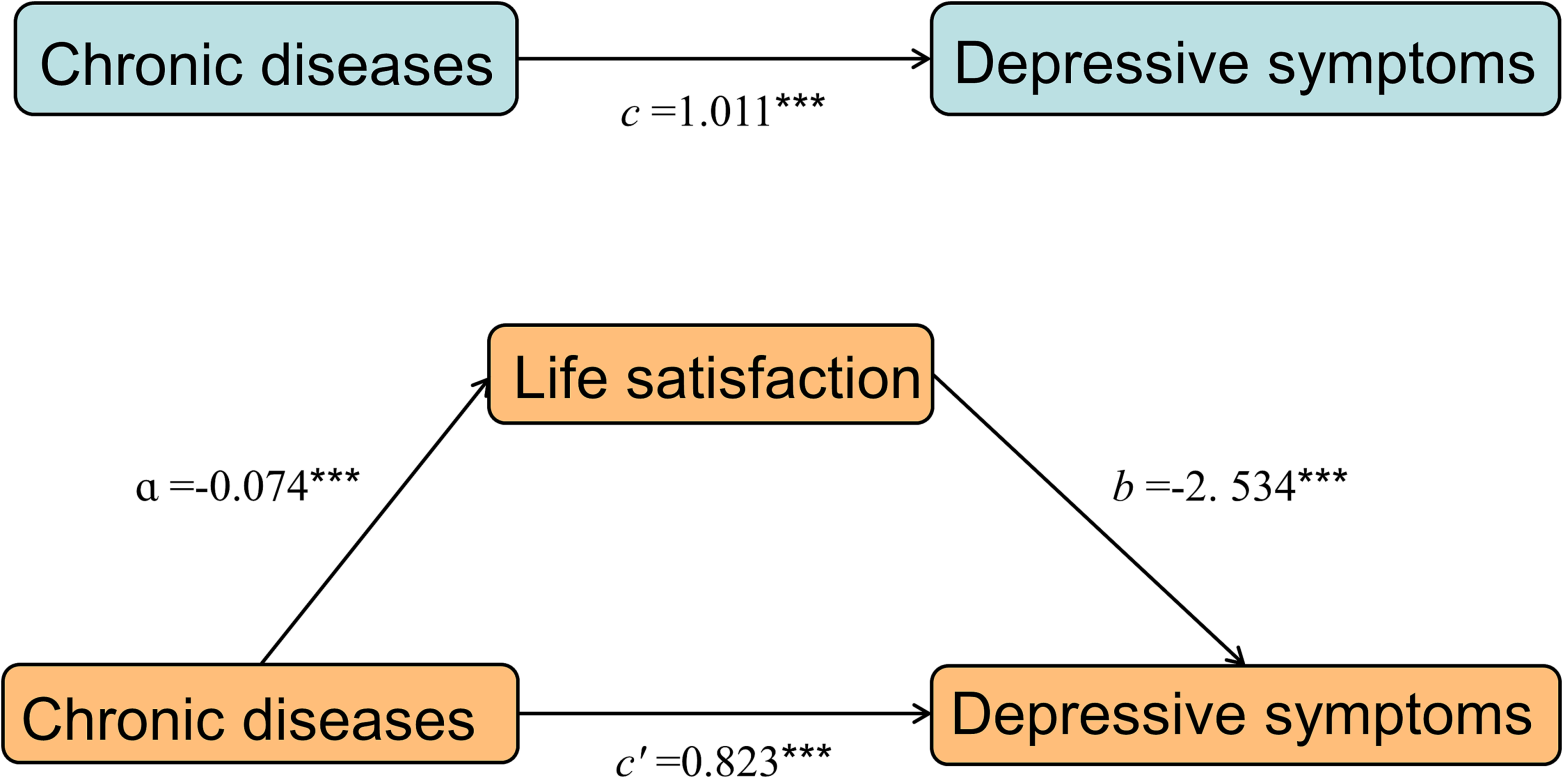
A mediating effect diagram. ***p < 0.001 a: Effect of the independent variable (chronic disease) on the mediating variable (life satisfaction). b: Effect of the mediating variable (life satisfaction) on the dependent variable (depressive symptoms). c: The total effect of chronic diseases on depressive symptoms. c’: The direct effect of chronic diseases.

After controlling all demographic and health-related variables, the results of the mediation effect analysis showed that the total effect of chronic diseases on depressive symptoms was path *c* = 1.011 (*p* < 0.001). The indirect path had the following parameters: path *a* = −0.074 (*p* < 0.001), path *b* = −2.534 (*p* < 0.001), the indirect effect of life satisfaction was (path *a* × path *b*) = 0.188 (*p* < 0.001), and bootstrap 95% confidence interval did not contain 0. It suggests the validity of the mediating effect of life satisfaction. The direct effect of chronic illnesses (path *c*’) was 0.823, which was still significant (*p* < 0.001), showing that the mediating effect was partial, and the extent of the mediating effect (*ab/c*) was 18.6% (Table 4, Figure 3).

**Table 4:** Intermediate effect test results.

Variables	*R* ^2^	*F*	*β*	*S. E*	*t*	*p*	*LLCI*	*ULCI*
Life satisfaction (Y)	0.015	14.354***						
Chronic diseases			−0.074	0.009	−8.163	<0.001	−0.092	−0.056
Depressive symptoms (Y)	0.189	200.259***						
Chronic disease			0.823	0.062	13.393	<0.001	0.703	0.944
Life satisfaction			−2.534	0.077	−33.131	<0.001	−2.684	−2.384
Total effect	0.074	77.162***	1.011	0.065	15.464	<0.001	0.883	1.393
Indirect effect			0.188	0.025			0.140	0.236

LLCI, lower limit of confidence interval; ULCI, upper limit of confidence interval.

Gender, age, marital status, education level, annual household income, smoking, and drinking as the control variable.

***p < 0.001.

### Moderating effect of living arrangements

**Figure 4:**
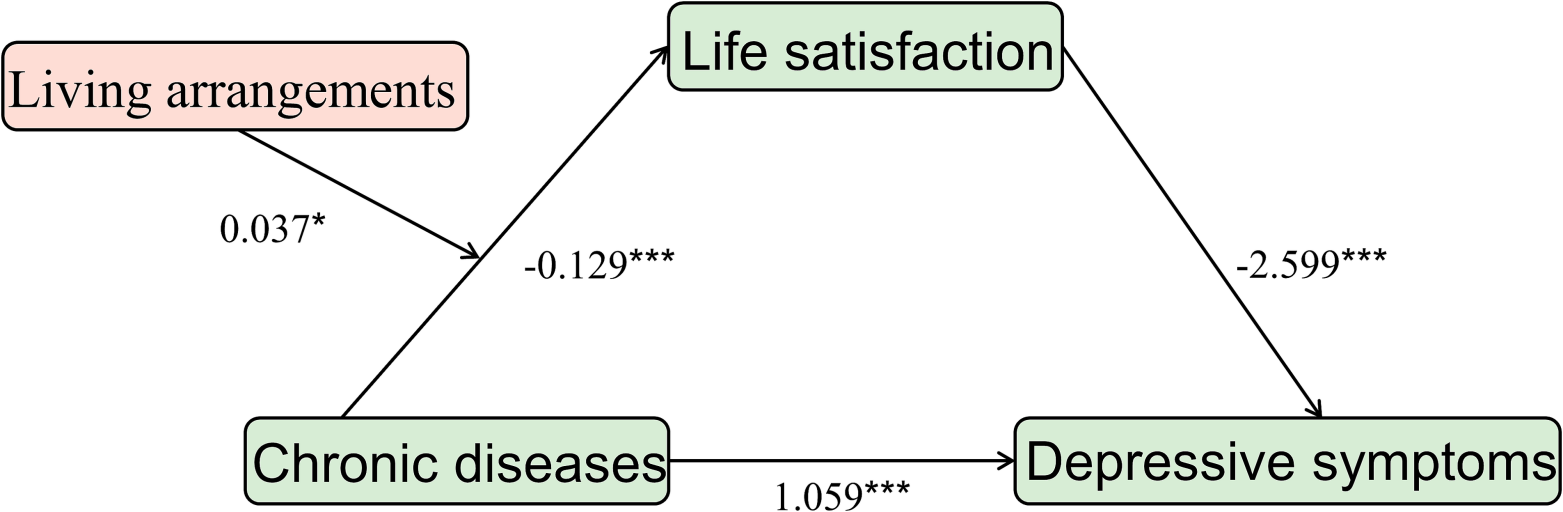
A moderated mediation model diagram. ***p < 0.001,*p < 0.05.

The moderated mediation analysis indicated that the living arrangements moderated the first half of the path *a* (=0.037, *p* < 0.05) in the mediation model after controlling for demographic and health-related factors. Living arrangements moderated the relationship between chronic diseases and life satisfaction. The interaction term between chronic diseases and living arrangements predicts life satisfaction (Table 5, Figure 4). However, it did not play a moderating role in the second half of path *b* (=0.042, *p* > 0.05) or the direct path (= − 0.162, *p* > 0.05) in the mediation model.

**Table 5:** Moderating effect test results.

Variables	*R* ^2^	*F*	*β*	*S. E*	*t*	*p*	*LLCI*	*ULCI*
Life satisfaction (Y)	0.015	11.737***						
Chronic diseases			−0.129	0.029	−4.513	<0.001	−0.185	−0.073
Living arrangements			−0.003	0.018	−0.176	0.860	−0.038	0.032
Chronic diseases × Living arrangements			0.037	0.018	2.021	0.043	0.001	0.072
Depressive symptoms (Y)	0.199	160.238***						
Chronic disease			1.059	0.019	5.523	<0.001	0.684	1.436
Life satisfaction			−2.599	0.245	−10.632	<0.001	−3.079	−2.120
Living arrangements			0.018	0.504	−0.036	0.972	−0.970	1.006
Chronic diseases × Living arrangements			−0.162	0.121	−1.330	0.184	−0.400	0.077
Life satisfaction × Living arrangements			0.042	0.152	0.278	0.790	−0.256	0.340

LLCI, lower limit of confidence interval; ULCI, upper limit of confidence interval.

Gender, age, marital status, education level, annual household income, smoking, and drinking as control variables.

***p < 0.001.

**Figure 5:**
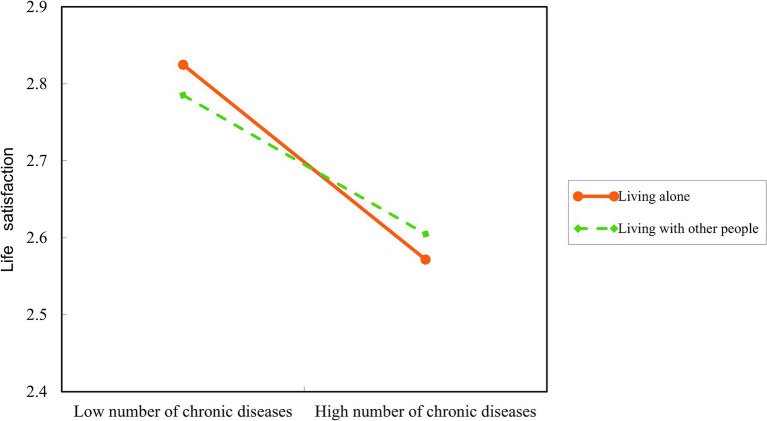
A simple slope test on the specific moderating effect of living arrangements.

A simple slope analysis of living arrangements was used to investigate to explore the effects of chronic diseases on life satisfaction among those living alone and living with other people. According to the results, with the increase in chronic disease number, the life satisfaction of people who live alone declined significantly more than that of those who live with others (simple slope, living alone: −0.13, *p* < 0.001, living with other people −0.10, *p* < 0.001) (Figure 5).

## Discussion

This study indicated that the population’s total prevalence of depressive symptoms was 30.3%. The prevalence of depressive symptoms is mostly higher than the results of existing studies (Jung et al., 2018; Alinaitwe et al., 2021), which may be due to differences in measurement tools and the severity of the disease. The presence of depressive symptoms is more possibly to be female, have a lower education level, and have lower annual household income than those without depressive symptoms. More than 49.6% had at least one chronic disease, and nearly 20.3% had at least two chronic diseases among those with depressive symptoms. These results are generally consistent with earlier studies (Liu et al., 2018; Li et al., 2019; Hu et al., 2022). Given that those who exhibit the aforementioned unfavorable characteristics are more likely to suffer from depressive symptoms, we need to promote mental health and pay attention to how chronic diseases affect them.

According to the results of this study, hypothesis 1 is confirmed, and there is a positive correlation between the prevalence of chronic diseases and depressive symptoms in middle-aged and older people. The severity of the depression symptoms increased with the number of chronic conditions, consistent with previous research (Seo et al., 2017; Bi et al., 2021; Zhang et al., 2022). Most chronic disease patients have diminished physical capacity, which negatively impacts everyday activities and lowers the quality of life. They might experience discomfort, anxiety, and even despair as they face challenges in their everyday lives (Tinetti et al., 2012). Some patients with chronic diseases need to accept care from family members and relatives, which adds to their psychological burden and makes them feel inferior. Another possibility is that people with chronic diseases put a significant financial strain on their families and themselves because of the lengthy treatment times and hefty medical costs. Patients who cannot take care of themselves depend on family members for care. Family members’ working hours and total family income will be reduced, and the financial burden on the family will increase (Dong et al., 2020). They were influenced by the traditional Chinese belief that most middle-aged and older adults should care for their children or grandchildren (Ma et al., 2021). Middle-aged and older people with chronic diseases who require care may feel like a burden and experience unpleasant feelings, including irritation, worry, pessimism, and despair. Therefore, enhancing chronic disease evaluation and management becomes a crucial component of preventing and treating depression in middle-aged and older people.

Hypothesis 2 is supported that the mediating effect results suggest that chronic disease can either directly impact the depressive symptoms of the middle-aged and elderly or indirectly affect their depressive symptoms through the effect of life satisfaction, which mediates the relationship between chronic diseases and depression symptoms to some extent. First, there is a direct positive predictive relationship between depressive symptoms and chronic illness. This suggests that an increase in the number of chronic diseases can put patients under additional stress. Middle-aged and older people may have more severe depressive symptoms. This study suggests that those aroused by stress typically have poor mental health (Ma et al., 2021). Second, chronic diseases can also indirectly impact depressive symptoms by lowering life satisfaction. According to earlier research, older people’s life satisfaction suffers when they have more concurrent chronic illnesses, which in turn cause depressive symptoms (Koivumaa-Honkanen et al., 2004; de Guzman et al., 2015). Chronic diseases and depressive symptoms might be connected to life satisfaction. A prior study found that older people’s life satisfaction might be decreased by less effective coping mechanisms brought on by stressful life experiences, promoting the mental health of middle-aged and older people by adopting positive stress-coping measures to ameliorate the adverse effects of stressful events (Ren et al., 2021). As a result, by successfully preventing and managing the development and progression of chronic diseases, we can not only reduce depressive symptoms in middle-aged and older people, but we can also prevent them from occurring by enhancing life satisfaction for these people, thereby enhancing their mental health.

It shows that living arrangements have a moderating effect on the association between chronic illness and life satisfaction. Based on the simple slope analysis findings, we discovered that while chronic diseases affected life satisfaction, their severity varied between middle-aged and older adults who lived with others and those who lived alone. Chronic diseases have a greater impact on the life satisfaction of middle-aged and older adults who live alone than those who live with others. A previous study has shown that living with a partner benefits role functioning in individuals with multiple chronic diseases. When middle-aged and older people living alone suffer from single or multiple chronic diseases (Müller et al., 2017), they do not have the daily care and moral support from their family members, and the pressure and solitude associated with the disease can impact their life satisfaction. Middle-aged and older adults have stronger subjective well-being and are happier with their lives when their family atmospheres are warm and harmonious and receive strong social support (Yu, 2017; Mao and Han, 2018). To increase their life satisfaction or improve chronic illness health management to lessen the incidence of depressive symptoms, it is advised to pay attention to the life satisfaction of middle-aged and older people living alone with chronic diseases.

### Practical implications

Based on the above analysis, we should focus on depressive symptoms in middle-aged and older people with chronic diseases, increase public awareness of these conditions, and enhance the diagnostic, management, and preventative control of these illnesses. Second, we should guide middle-aged and older people with chronic diseases to maintain better living conditions and improve their life satisfaction by restoring their independent activities and living ability through standardized treatment, rehabilitation exercises, health education, and social support, reducing the impact of diseases on their lives. Third, we should care for middle-aged and elderly who live alone, understand their health and psychological needs, and strengthen their contact and social support with their families to enhance their health and reduce depressive symptoms.

### Limitations

There are several limitations to this study. First, this study focused mechanisms of the effect of the number of chronic conditions on depressive symptoms and did not explore different types of chronic degenerative diseases or focus on the severity of the disease. This section will be further analyzed in future studies. Second, the results of the current study revealed that life satisfaction partially mediated the association between chronic illnesses and depressive symptoms, suggesting the possibility of other mediating factors. Third, this study used cross-sectional data from 2018, which has limitations in illustrating the causal link between chronic illnesses and depressive symptoms. Consequently, we will consider utilizing longitudinal data for analysis in future research.

## Conclusion

In the current research, chronic diseases significantly affected middle-aged and older people’s depressive symptoms, and the more chronic diseases a person had, the more severe their depressive symptoms were. Chronic diseases had a predictive influence on life satisfaction in middle-aged and older people who live alone, and life satisfaction mediated the association between chronic diseases and depressive symptoms.

## Data availability statement

Publicly available datasets were analyzed in this study. This data can be found at: http://charls.pku.edu.cn/en.

## Ethics statement

The studies involving humans were approved by the Institutional Review Board at Peking University. The studies were conducted in accordance with the local legislation and institutional requirements. The participants provided their written informed consent to participate in this study.

## Author contributions

ZS: Conceptualization, Data curation, Investigation, Methodology, Writing – original draft. YQL: Data curation, Investigation, Methodology, Writing – original draft. DX: Data curation, Investigation, Methodology, Writing – review & editing. YZ: Data curation, Investigation, Methodology, Writing – review & editing. YPL: Conceptualization, Supervision, Writing – review & editing. BZ: Conceptualization, Supervision, Writing – review & editing. YD: Conceptualization, Funding acquisition, Methodology, Resources, Supervision, Writing – review & editing.

## Abbreviations

CHARLS: China Health and Retirement Longitudinal Study

## References

[ref1] Aiken L. S. West S. G. (1991). Multiple regression: testing and interpreting interactions. Newbury Park. Sage Publications, Inc.

[ref2] Alinaitwe R. Birungi C. Bangirana P. Nakasujja N. (2021). Prevalence and factors associated with depressive illness in patients with tuberculosis in Mulago hospital, Kampala- Uganda: a cross sectional study. J. Psychosom. Res. 149:110591. doi: 10.1016/j.jpsychores.2021.110591, PMID: 34390942

[ref3] Anderies J. M. (2014). Embedding built environments in social–ecological systems: resilience-based design principles. Build. Res. Inf. 42, 130–142. doi: 10.1080/09613218.2013.857455

[ref4] Bi Y. H. Pei J. J. Hao C. Yao W. Wang H. X. (2021). The relationship between chronic diseases and depression in middle-aged and older adults: a 4-year follow-up study from the China health and retirement longitudinal study. J. Affect. Disord. 289, 160–166. doi: 10.1016/j.jad.2021.04.032, PMID: 33984686

[ref5] Chen H. Mui A. C. (2014). Factorial validity of the center for epidemiologic studies depression scale short form in older population in China. Int. Psychogeriatr. 26, 49–57. doi: 10.1017/s1041610213001701, PMID: 24125553

[ref6] Chokkanathan S. Mohanty J. (2017). Health, family strains, dependency, and life satisfaction of older adults. Arch. Gerontol. Geriatr. 71, 129–135. doi: 10.1016/j.archger.2017.04.001, PMID: 28432920

[ref7] de Guzman A. B. Jurado J. B. N. Juson A. J. A. (2015). Examining the structural relationship of chronic illness, physical function, life satisfaction, and social support in the development of depression among Filipino elderly in institutionalized settings. Educ. Gerontol. 41, 193–206. doi: 10.1080/03601277.2014.918836

[ref8] Dong X. Chen L. Xu Z. Xu X. (2020). An assessment of the economic burden of senile chronic diseases in China based on China health and retirement longitudinal survey. Expert Rev. Pharmacoecon. Outcomes Res. 20, 305–312. doi: 10.1080/14737167.2020.1688661, PMID: 31675261

[ref9] Drop B. Janiszewska M. Barańska A. Kanecki K. Nitsch-Osuch A. Bogdan M. (2018). Satisfaction with life and adaptive reactions in people treated for chronic obstructive pulmonary disease. Adv. Exp. Med. Biol. 1114, 41–47. doi: 10.1007/5584_2018_242, PMID: 30051324

[ref10] Gao N. Yao J. (2020). The effects of living arrangement on the depression of older adults. J. Nanjing Med. Univ. 2, 140–145. doi: 10.7655/NYDXBSS20200208

[ref11] Hayes A. F. (2013). Introduction to mediation, moderation, and conditional process analysis: a regression-based approach. Guilford Press. New York.

[ref12] Hu S. X. X. Lei W. I. Chao K. K. Hall B. J. Chung S. F. (2016). Common chronic health problems and life satisfaction among Macau elderly people. Int. J. Nurs. Sci. 3, 367–370. doi: 10.1016/j.ijnss.2016.10.004

[ref13] Hu J. Zheng X. Shi G. Guo L. (2022). Associations of multiple chronic disease and depressive symptoms with incident stroke among Chinese middle-aged and elderly adults: a nationwide population-based cohort study. BMC Geriatr. 22:660. doi: 10.1186/s12877-022-03329-4, PMID: 35953770 PMC9373457

[ref14] Jung S. Lee S. Lee S. Bae S. Imaoka M. Harada K.. (2018). Relationship between physical activity levels and depressive symptoms in community-dwelling older Japanese adults. Geriatr. Gerontol. Int. 18, 421–427. doi: 10.1111/ggi.13195, PMID: 29052928

[ref15] Kandapan B. Pradhan J. Pradhan I. (2023). Living arrangement of Indian elderly: a predominant predictor of their level of life satisfaction. BMC Geriatr. 23:88. doi: 10.1186/s12877-023-03791-8, PMID: 36765271 PMC9921119

[ref16] Koivumaa-Honkanen H. Kaprio J. Honkanen R. Viinamäki H. Koskenvuo M. (2004). Life satisfaction and depression in a 15-year follow-up of healthy adults. Soc. Psychiatry Psychiatr. Epidemiol. 39, 994–999. doi: 10.1007/s00127-004-0833-6, PMID: 15583908

[ref17] Kooshiar H. Yahaya N. Hamid T. A. Abu Samah A. Sedaghat Jou V. (2012). Living arrangement and life satisfaction in older Malaysians: the mediating role of social support function. PLoS One 7:e43125. doi: 10.1371/journal.pone.0043125, PMID: 22912806 PMC3422218

[ref18] Li L. Ma M. Peng H. Yan Z. Wang M. Hu M.. (2021). Prevalence and associated factors of depressive symptoms in China's rural elderly. Chinese Gen. Pract. 24:3432. doi: 10.12114/j.issn.1007-9572.2021.00.577

[ref19] Li H. Zheng D. Li Z. Wu Z. Feng W. Cao X.. (2019). Association of depressive symptoms with incident cardiovascular diseases in middle-aged and older Chinese adults. JAMA Netw. Open 2:e1916591. doi: 10.1001/jamanetworkopen.2019.16591, PMID: 31800066 PMC6902756

[ref20] Liu Q. Cai H. Yang L. H. Xiang Y. B. Yang G. Li H.. (2018). Depressive symptoms and their association with social determinants and chronic diseases in middle-aged and elderly Chinese people. Sci. Rep. 8:3841. doi: 10.1038/s41598-018-22175-2, PMID: 29497126 PMC5832867

[ref21] Ma L. Tang Z. Sun F. Diao L. Li Y. Wang J.. (2015). Risk factors for depression among elderly subjects with hypertension living at home in China. Int. J. Clin. Exp. Med. 8, 2923–2928. PMID: 25932256 PMC4402903

[ref22] Ma Y. Xiang Q. Yan C. Liao H. Wang J. (2021). Relationship between chronic diseases and depression: the mediating effect of pain. BMC Psychiatry 21:436. doi: 10.1186/s12888-021-03428-3, PMID: 34488696 PMC8419946

[ref23] Malhi G. S. Mann J. J. (2018). Depression. Lancet 392, 2299–2312. doi: 10.1016/s0140-6736(18)31948-2 30396512

[ref24] Mao X. Han W. J. (2018). Living arrangements and older adults' psychological well-being and life satisfaction in China: does social support matter? Fam. Relat. 67, 567–584. doi: 10.1111/fare.12326

[ref25] Mei S. Qin Z. Yang Y. Gao T. Ren H. Hu Y.. (2021). Influence of life satisfaction on quality of life: mediating roles of depression and anxiety among cardiovascular disease patients. Clin. Nurs. Res. 30, 215–224. doi: 10.1177/1054773820947984, PMID: 32757768

[ref26] Mendez Y. P. Ralston P. A. Wickrama K. Bae D. Young-Clark I. Ilich J. Z. (2018). Lower life satisfaction, active coping and cardiovascular disease risk factors in older African Americans: outcomes of a longitudinal church-based intervention. J. Behav. Med. 41, 344–356. doi: 10.1007/s10865-017-9909-0, PMID: 29357010 PMC5924620

[ref27] Müller F. Hagedoorn M. Tuinman M. A. (2017). Chronic multimorbidity impairs role functioning in middle-aged and older individuals mostly when non-partnered or living alone. PLoS One 12:e0170525. doi: 10.1371/journal.pone.0170525, PMID: 28151967 PMC5289430

[ref28] Pan A. Keum N. Okereke O. I. Sun Q. Kivimaki M. Rubin R. R.. (2012). Bidirectional association between depression and metabolic syndrome: a systematic review and meta-analysis of epidemiological studies. Diabetes Care 35, 1171–1180. doi: 10.2337/dc11-2055 22517938 PMC3329841

[ref29] Park E. J. Sohn H. S. Lee E. K. Kwon J. W. (2014). Living arrangements, chronic diseases, and prescription drug expenditures among Korean elderly: vulnerability to potential medication underuse. BMC Public Health 14:1284. doi: 10.1186/1471-2458-14-1284 25516064 PMC4301451

[ref30] Read J. R. Sharpe L. Modini M. Dear B. F. (2017). Multimorbidity and depression: a systematic review and meta-analysis. J. Affect. Disord. 221, 36–46. doi: 10.1016/j.jad.2017.06.009 28628766

[ref31] Ren Q. Jiang C. Jiang S. (2021). Stressful life events and life satisfaction among Chinese older adults: the role of coping styles. Healthcare 9:1620. doi: 10.3390/healthcare9121620, PMID: 34946345 PMC8701379

[ref32] Rosella L. C. Fu L. Buajitti E. Goel V. (2019). Death and chronic disease risk associated with poor life satisfaction: a population-based cohort study. Am. J. Epidemiol. 188, 323–331. doi: 10.1093/aje/kwy245, PMID: 30371732 PMC6357802

[ref33] Seo J. Choi B. Kim S. Lee H. Oh D. (2017). The relationship between multiple chronic diseases and depressive symptoms among middle-aged and elderly populations: results of a 2009 korean community health survey of 156,747 participants. BMC Public Health 17:844. doi: 10.1186/s12889-017-4798-2, PMID: 29070021 PMC5657127

[ref34] Shad B. Ashouri A. Hasandokht T. Rajati F. Salari A. Naghshbandi M.. (2017). Effect of multimorbidity on quality of life in adult with cardiovascular disease: a cross-sectional study. Health Qual. Life Outcomes 15:240. doi: 10.1186/s12955-017-0820-8, PMID: 29221456 PMC5723093

[ref35] Smorti M. Guarnieri S. Bergesio F. Perfetto F. Cappelli F. (2016). Anxiety and depression among amyloid light-chain cardiac amyloidosis patients: the role of life satisfaction. Eur. J. Cardiovasc. Nurs. 15, 269–275. doi: 10.1177/1474515114566737, PMID: 25601945

[ref36] Srivastava S. Debnath P. Shri N. Muhammad T. (2021). The association of widowhood and living alone with depression among older adults in India. Sci. Rep. 11:21641. doi: 10.1038/s41598-021-01238-x, PMID: 34737402 PMC8568934

[ref37] Steca P. Greco A. Monzani D. Politi A. Gestra R. Ferrari G.. (2013). How does illness severity influence depression, health satisfaction and life satisfaction in patients with cardiovascular disease? The mediating role of illness perception and self-efficacy beliefs. Psychol. Health 28, 765–783. doi: 10.1080/08870446.2012.759223, PMID: 23343116

[ref38] Tanrıverdi Ö. Korkmaz M. (2021). The relationship between self-effıcacy and life satisfaction in patients with chronic obstructive pulmonary disease. J. Public Health 31, 1–8. doi: 10.1007/s10389-021-01576-0

[ref39] Tinetti M. E. Fried T. R. Boyd C. M. (2012). Designing health care for the most common chronic condition--multimorbidity. JAMA 307, 2493–2494. doi: 10.1001/jama.2012.5265, PMID: 22797447 PMC4083627

[ref40] Violán C. Bejarano-Rivera N. Foguet-Boreu Q. Roso Llorach A. Pons-Vigués M. Martin Mateo M.. (2016). The burden of cardiovascular morbidity in a European Mediterranean population with multimorbidity: a cross-sectional study. BMC Fam. Pract. 17:150. doi: 10.1186/s12875-016-0546-4, PMID: 27809772 PMC5093992

[ref41] Wang J. Xu J. Nie Y. Pan P. Zhang X. Li Y.. (2022). Effects of social participation and its diversity, frequency, and type on depression in middle-aged and older persons: evidence from China. Front. Psych. 13:825460. doi: 10.3389/fpsyt.2022.825460, PMID: 35546944 PMC9085245

[ref42] Wister A. Kendig H. Mitchell B. Fyffe I. Loh V. (2016). Multimorbidity, health and aging in Canada and Australia: a tale of two countries. BMC Geriatr. 16:163. doi: 10.1186/s12877-016-0341-z, PMID: 27663198 PMC5035492

[ref43] World Health Organization (2017). Depression and other common mental disorders: global health estimates. America. World Health Organization

[ref44] Xiu-Ying H. Qian C. Xiao-Dong P. Xue-Mei Z. Chang-Quan H. (2012). Living arrangements and risk for late life depression: a meta-analysis of published literature. Int. J. Psychiatry Med. 43, 19–34. doi: 10.2190/PM.43.1.b, PMID: 22641928

[ref45] Yu Z. (2017). Influence of intergenerational support on the life satisfaction of the elderly and the differences between urban and rural areas: based on analysis of 7669 samples from CHARLS. J. Hunan Univ. Agric. 18, 62–69.

[ref46] Zhang C. Chang Y. Yun Q. Lu J. Zheng X. Xue Y.. (2022). The impact of chronic diseases on depressive symptoms among the older adults: the role of sleep quality and empty nest status. J. Affect. Disord. 302, 94–100. doi: 10.1016/j.jad.2022.01.090, PMID: 35085671

